# Analysis of influencing factors and structural changes in hospitalization expenses for heart failure patients under the DRG payment system reform

**DOI:** 10.3389/fpubh.2026.1810483

**Published:** 2026-05-05

**Authors:** Danqi Huang, Hongda Gao, He Chen, Jinping Huang, Jinmin Zhao

**Affiliations:** 1 School of Information and Management, Guangxi Medical University, Nanning, China; 2 School of Public Health and Management, Guangxi University of Chinese Medicine, Nanning, China

**Keywords:** DRG payment system reform, heart failure, hospitalization expenses, influencing factors, structural change

## Abstract

**Objective:**

This study aimed to explore the influencing factors and structural changes in hospitalization expenses for heart failure (HF) patients under the Diagnosis-Related Group (DRG) payment system reform, providing a scientific basis for hospitals to control the hospitalization costs of HF patients.

**Methods:**

A total of 4,791 hospitalized HF patients from a secondary Grade-A hospital in Liuzhou City between January 1, 2015, and July 31, 2025, were selected as the study subjects. Factors such as the timing of DRG implementation were grouped and included in the analysis. Univariate analysis and multiple linear regression were employed to analyze the influencing factors of total hospitalization expenses and out-of-pocket payments. The degree of structural change and novel gray relational analysis were used to examine the trends and correlations in the structure of hospitalization expenses for HF patients.

**Results:**

The total hospitalization expenses of HF patients were mainly influenced by the type of medical insurance, number of visits, gender, age, length of stay, number of other diagnosed diseases, diabetes, surgery level, and discharge method (*P* < 0.05). Out-of-pocket payments were primarily affected by the payment method group, type of medical insurance, number of visits, gender, marital status, admission route, length of stay, and surgery level (*P* < 0.05). From 2015 to 2025, the treatment fee had the largest Variation of Structural Value (VSV) at 0.1663, showing a positive change, while the drug fee contributed the most to the structural change at 34.06%. The novel gray relational analysis indicated that the top two associated items were diagnosis fees (0.8797) and drug fees (0.7647), meaning that treatment, diagnosis, and drug fees were the main factors affecting the structural changes in hospitalization expenses.

**Conclusion:**

The DRG payment system can effectively reduce the hospitalization expenses of HF patients, alleviating their disease burden. When controlling costs under the DRG payment system, comprehensive consideration should be given to influencing factors such as the patient's number of visits, length of stay, surgery level, gender, age, and number of other diagnosed diseases to effectively reduce hospitalization expenses. By utilizing DRG payments and medical insurance policies to manage and control diagnosis and drug fees of heart failure, hospitalization costs can be controlled, and the cost structure optimized.

## 1 Introduction

Heart Failure (HF) is a severe manifestation or advanced stage of various cardiac diseases, characterized by high mortality and readmission rates. The prevalence of HF among adults in developed countries ranges from 1.0 to 2.0% (1). In 2021, approximately 55.5 million people worldwide were affected by HF (2). In the United States, there are about 6 million HF patients (3), projected to exceed 8 million by 2030, with annual direct medical costs for HF amounting to approximately $30.7 billion (4). In Australia, over 61,000 adults are diagnosed with HF annually, with the annual cost of managing HF in the community around $0.9 billion, rising to nearly $2.7 billion when additional inpatient care costs are included (5). In China, the prevalence of HF among adults aged ≥35 is 1.3%, with about 13.7 million HF patients (1). In 2023, China admitted 14.29 million HF inpatients, with hospitalization expenses reaching approximately ¥22.98 billion (6). As a complex clinical syndrome, HF imposes a significant economic burden, primarily due to its high prevalence and hospitalization needs. With China's aging population, the prevalence and treatment costs of HF are expected to rise significantly, increasing the economic burden (7).

The economic burden caused by HF has become a key focus in the global healthcare field. Studies indicate that hospitalization expenses account for 15%−92% of the total medical costs for HF (8), constituting the main component of the HF economic burden. Numerous scholars have conducted research on the factors influencing hospitalization expenses for HF patients. Studies on HF patient hospitalization expenses in Tianjin of China, showed that higher hospital levels, longer hospital stays, and surgical treatment were associated with higher costs, while female patients had lower expenses than males (9). Research in Xinjiang of China found that length of stay, comorbidities or complications, and treatment methods were influencing factors (10). In Shanxi Province of China, the main factors affecting total hospitalization expenses for HF cases were length of stay, followed by whether surgery was performed, number of rescues, occupation, number of hospitalizations, age, payment method, outcome, and gender (11). Other studies have shown that drug, material, treatment, and examination fees are factors influencing the internal structural changes of HF hospitalization expenses (11–13).

There are numerous studies both domestically and internationally on measures to control hospitalization costs for heart failure. For example, medication management to control costs—research from Spain indicates that initiating Sacubitril/valsartan at the onset of heart failure hospitalization is cost-effective (14). Alternatively, payment policies and incentives also play a role, America's Hospital Readmissions Reduction Program (HRRP) thereby has directly reduced Medicare expenditures substantially, through penalties assessed to hospitals and by decreasing expenditures for patients rehospitalized early after index admission (15). China's DRG payment reform aims to change how medical insurance pays hospitals, incentive them to actively control costs and reduce unnecessary medical practices. Domestic studies suggest that China's DRG payment system can effectively control hospitalization expenses, demonstrating significant cost-reduction and burden-alleviation effects, primarily by lowering material and drug fees (16), including controlled hospitalization expenses and length of stay for patients with cardiovascular and cerebrovascular diseases (17). Both DRG and DIP payment methods have a regulatory effect on the hospitalization expense structure of cardiovascular disease patients (18).

Guangxi, as an economically underdeveloped region in western China with a concentrated ethnic minority population, and Liuzhou City, as one of the first pilot cities for the DRG payment system in Guangxi, have seen limited research on HF hospitalization expenses from a DRG payment perspective. This study will investigate the hospitalization expenses of HF patients in a secondary Grade-A general hospital in Liuzhou City, Guangxi, expanding the research perspective to explore the influencing factors and structural changes in HF hospitalization expenses under the DRG payment system reform, aiming to reduce the economic burden of HF.

## 2 Data sources

Data were derived from the front page of inpatient medical records of HF patients at a secondary Grade-A general hospital in Liuzhou City, Guangxi, China. Study subjects were selected based on inclusion and exclusion criteria. Inclusion criteria: ① Admitted between January 1, 2015, and July 31, 2025. ② Primary diagnosis with specific code I50.9 for cardiac system diseases according to the national CHS-DRG grouping scheme (version 1.0). ③ Complete records in electronic medical records, including the front page, patient disease diagnosis and treatment records, and clinical characteristic information. Exclusion criteria: ① Extreme values: length of stay less than 1 day (equal to 0) or greater than 60 days. ② Incomplete electronic medical record information or obvious logical errors, such as admission route is empty. Finally, 4,791 valid cases were included. Research variables and their assignments were determined, as shown in Table 1. These mainly include patients' basic demographic information, basic hospitalization and treatment details, total hospitalization expenses, out-of-pocket payments, and detailed itemized expenses. Total hospitalization expenses were subdivided into seven categories: comprehensive medical service fees, diagnosis fees, treatment fees, drug fees, blood and blood product fees, material fees, and other fees.

**Table 1:** Variable selection and assignment.

Variable names	Code	Assignment
Payment method group	X1	Phase 1: “Non-DRG Period,” assigned a value of “1”; Phase 2: “Liuzhou DRG Period,” assigned a value of “2”; Phase 3: “Guangxi DRG Period,” assigned a value of “3”
Type of medical insurance	X2	Uninsured = 1, Urban-Rural Resident Medical Insurance = 2, Urban Employee Medical Insurance = 3
Number of visits	X3	1 = 1, 2 = 2, 3 = 3, ≥4 = 4
Gender	X4	Male = 1, Female = 2
Age (years)	X5	1–60 = 1, 61–65 = 2, 66–70 = 3, 71–75 = 4, 76–80 = 5, 81–85 = 6, 86–90 = 7, >90 = 8
Nationality	X6	Han=1, Zhuang=2, Other ethnic groups=3
Marital status	X7	Single = 1, Married = 2, Divorced = 3, Widowed = 4, Other = 5
Admission route	X8	Emergency = 1, Outpatient = 2
Length of stay (days)	X9	≤ 3 = 1, 4–6 = 2, 7–9 = 3, >9 = 4
Number of other diagnosed diseases	X10	1–3 = 1, 4–6 = 2, 7–9 = 3, 10–12 = 4, 13–15 = 5, ≥16 = 6
Hypertension	X11	Yes = 1, No = 0
Diabetes	X12	Yes = 1, No = 0
Surgery level	X13	No surgery = 0, Level 1 surgery = 1, Level 2 surgery = 2, Level 3 surgery = 3, Uncertain level = 4
Discharge method	X14	Transfer by doctor's order = 1, Discharge by doctor's order = 2, Discharge not by doctor's order = 3, Death = 4, Other = 5
Total hospitalization expenses	Y1	Total hospitalization expenses adjusted according to CPI and log-transformed
Out-of-pocket payments	Y2	Out-of-pocket payments adjusted according to CPI and log-transformed

Details of the coding and assignment processing for each variable are shown in Table 1. Among them, the variable “Payment Method Group” was grouped based on the period when the sample hospital implemented DRG: Phase 1 covered the period before DRG implementation (January 1, 2015–August 31, 2017), referred to as the “Non-DRG Period”; Phase 2 covered the period of Liuzhou (municipal) DRG payment implementation (September 1, 2017–June 30, 2022), referred to as the “Liuzhou DRG Period”; Phase 3 covered the period of Guangxi (provincial) DRG payment implementation (July 1, 2022–July 31, 2025), referred to as the “Guangxi DRG Period” Total hospitalization expenses refer to all actual medical costs incurred during hospitalization. Out-of-pocket payments refer to the amount actually paid by the patient after medical insurance reimbursement of the total hospitalization expenses.

This study has been approved by the Medical Ethics Committee of Guangxi Medical University.

## 3 Research methods

### 3.1 Standardization

To eliminate the impact of inflation and other economic factors on hospitalization expense values and enhance data comparability, hospitalization expenses were adjusted based on the Chinese Consumer Price Index (CPI) (19). Using 2015 as the base period, hospitalization expenses for other discharge years were standardized. Log-transformed data were then used for statistical correlation analysis.

### 3.2 Degree of structural change analysis

This method analyzes the overall characteristics and dynamic trends of expense composition, comprehensively reflecting the dynamic changes in the internal expense structure (20). Specific steps:


$$\begin{array}{l} \text{①}\;\mathrm{Structural}\;\mathrm{Variation}\;\mathrm{Value}\;\left(\mathrm{VSV}\right)=\mathrm{Xi}1-\mathrm{Xi}0, \end{array}$$ (1)

where i represents the expense item number, Xi0 is the proportion of the i-th expense item at the beginning of the period, and Xi1 is the proportion at the end of the period. VSV > 0 indicates a positive change (increasing proportion), VSV < 0 indicates a negative change (decreasing proportion).


$$\begin{array}{l} \text{②}\;\mathrm{Degree}\;\mathrm{of}\;\mathrm{Structural}\;\mathrm{Variation}\;\left(\mathrm{DSV}\right)=\Sigma |\mathrm{VSV}| \end{array}$$ (2)

A larger DSV indicates greater structural fluctuation during the period.


$$\begin{array}{lll} \text{③}\;\mathrm{Contribution}\;\mathrm{Rate}\;\mathrm{of}\;\mathrm{Structural}\;\mathrm{Variation}\;\left(\mathrm{CRSV}\right) & = & \left|\mathrm{VSV}\right|\\ \;/\mathrm{DS}{\text{V}}^{*}100\%. \end{array}$$ (3)

### 3.3 Novel gray relational analysis

Gray relational analysis can effectively reflect the intrinsic connection between hospitalization expenses and the development trends of various itemized expenses. The novel gray relational analysis is an improved version that reduces the steps for dimensionless data processing. In this study, the average per-admission total hospitalization expenses from 2015 to 2025 served as the reference sequence X0(k), and the average per-admission expenses for each item served as comparative sequences Xi(k). Specific steps:

① Calculate the absolute difference between the reference sequence and each comparative sequence (difference sequence):


$$\begin{array}{l} \Delta \text{i}\left(\text{k}\right)=|\mathrm{Xi}\left(\text{k}\right)-\text{X}0\left(\text{k}\right)|, \end{array}$$ (4)

where K ranges from 1 to 11, representing the years 2015 to 2025; i represents the i-th expense item. Find the maximum and minimum differences (Δmax, Δmin) from Δi(K).

② Calculate each correlation coefficient:


$$\begin{array}{l} \xi \text{i}\left(\text{k}\right)=\left(\Delta \min+\rho^{*}\Delta \text{max}\right)/\left(\Delta \text{i}\left(\text{k}\right)+\rho^{*}\Delta \text{max}\right), \end{array}$$ (5)

with ρ = 0.5.

③ Calculate the relational degree and list the relational order:


$$\begin{array}{l} \gamma \text{i}=1/\text{N}\sum \xi \text{i}\left(\text{k}\right), \end{array}$$ (6)

where N = 11, representing the 11 natural years (21).

### 3.4 Statistical methods

Microsoft Excel 2010 (Microsoft Corporation, Redmond, WA, USA) was used to create and input data into the database. IBM SPSS Statistics 26.0 (IBM Corporation, Armonk, NY, USA) and Stata 17.0 (StataCorp LLC, College Station, TX, USA) were used for data analysis. The degree of structural change and novel gray relational analysis were employed to analyze the trends and correlations in the hospitalization expense structure of HF patients. The normality of total hospitalization expenses and out-of-pocket payments was tested using the one-sample Kolmogorov–Smirnov test, and the results did not meet the assumption of normal distribution (*P* < 0.05). Therefore, measurement data in this study were described using median, interquartile range, and frequency (percentage). With payment method group, type of medical insurance, number of visits, gender, age, ethnicity, marital status, admission route, length of stay, number of other diagnosed diseases, hypertension, diabetes, surgery level, and discharge method as independent variables, and hospitalization expenses (log-transformed total hospitalization expenses and out-of-pocket payments to approximate normality) as dependent variables, univariate analysis of influencing factors on hospitalization expenses was conducted using the Mann–Whitney *U* test for two-group comparisons and the Kruskal–Wallis *H* test for three or more group comparisons. Statistically significant indicators from the univariate analysis were used as independent variables in a multiple linear regression analysis for multivariate analysis to explore the influencing factors of hospitalization expenses. The statistical significance level was set at α = 0.05.

## 4 Results

### 4.1 Basic patient information

This study included 4,791 hospitalized HF patients: 2,408 males, 86.79% aged over 60, 78.90% of Zhuang ethnicity, and 90.84% married. 76.04% of patients used Urban-Rural Resident Basic Medical Insurance for payment, 54.64% were first-time visits, and 73.83% were admitted via outpatient clinics. Length of stay was concentrated between 4 and 9 days, accounting for 78.42%. The number of other diagnosed diseases was concentrated between 7 and 12 types, accounting for 57.69%. Among the 4,791 patients, 53.16% had hypertension, and 19.56% had diabetes, 81.74% did not undergo surgical treatment, and 82.17% were discharged following doctor's orders (Table 2).

**Table 2:** Univariate analysis of factors affecting total hospitalization expenses and out-of-pocket payments and basic conditions of HF patients in sample hospitals from 2015 to 2025.

Variable categories	*N*	Proportion (%)	Total hospitalization expenses			Out-of-pocket payments		
			M (P25, P75)/yuan	*Z/H*	*P-*value	M (P25, P75)/yuan	*Z/H*	*P-*value
Payment method group								
“Non-DRG Period”	188	3.92	4,240.21 (3,399.05, 5,818.08)	100.682	< 0.001	4,240.21 (3,399.05, 5,818.08)	926.91	< 0.001
“Liuzhou DRG Period”	2,185	45.61	4,359.89 (3,621.33, 5,432.12)			1,504.78 (865.55, 3,450.07)		
“Guangxi DRG Period”	2,418	50.47	4,000.19 (2,979.01, 5,805.07)			837.1 (0, 1,495.33)		
Type of medical insurance								
Uninsured	551	11.50	4,494.37 (3,502.88, 6,954.55)	58.83	< 0.001	3,719.06 (1,516.49, 5,325.79)	512.175	< 0.001
Urban-Rural Resident Medical Insurance	3,643	76.04	4,175.24 (3,262.67, 5,490.61)			1,231.29 (219.93, 1,947.8)		
Urban Employee Medical Insurance	597	12.46	4,159.63 (3,188.5, 5,506.47)			645.17 (0, 1,153.1)		
Number of visits								
1	2,618	54.64	4,310.01 (3,394.1, 5,764.44)	49.108	< 0.001	1,471.65 (463.18, 3,443.53)	300.078	< 0.001
2	876	18.28	4,199.35 (3,237.54, 5,789.48)			1,171.88 (153.28, 1,894.62)		
3	458	9.56	3,975.87 (3,035.2, 5,210.17)			1,020.1 (136.63, 1,541.65)		
≥4	839	17.51	4,043.46 (3,144.22, 5,198.92)			814.5 (0, 1,339.84)		
Gender								
Male	2,408	50.26	4,288.9 (3,254.11, 5,794.61)	−2.53	0.011	1,072.17 (66.89, 2,132.63)	−6.537	< 0.001
Female	2,383	49.74	4,137.49 (3,292.39, 5,398.08)			1,328.97 (565.11, 2,288.33)		
Age (years)								
1–60	633	13.21	4,110.61 (2,996.61, 5,894.31)	35.718	< 0.001	1,060.85 (161.34, 2,268.98)	19.523	0.007
61–65	381	7.95	4,305.78 (3,274.74, 6,064.97)			1,277.76 (199.19, 2,966.1)		
66–70	715	14.92	4,456.78 (3,496.39, 6,004.09)			1,154.4 (84, 2,268.02)		
71–75	801	16.72	4,209.45 (3,377.39, 5,662.4)			1,188.12 (34.8, 2,175.13)		
76–80	853	17.80	4,211.36 (3,259.99, 5,462.7)			1,291.88 (522, 2,415.98)		
81–85	869	18.14	4,209.46 (3,316.96, 5,441.05)			1,254.71 (523.93, 2,077.42)		
86–90	407	8.50	4,006 (3,176.67, 5,036.13)			1,289.69 (222.7, 2,033.35)		
>90	132	2.76	3,873.32 (3,022.36, 5,028.97)			1,162.37 (786.4, 1,853.67)		
Nationality								
Han	993	20.73	4,153.03 (3,143, 5,711.95)	3.357	0.187	1,057.82 (130.53, 1,728.4)	53.055	< 0.001
Zhuang	3,780	78.90	4,209.95 (3,311.37, 5,557.91)			1,282.6 (240.72, 2,505.91)		
Other ethnic groups	18	0.38	4,637.14 (3,236.25, 5,894.36)			45.6 (0, 1,184.33)		
Marital status								
Single	209	4.36	4,237.78 (3,200.34, 5,194.04)	5.125	0.275	72.6 (0, 901.91)	105.374	< 0.001
Married	4,352	90.84	4,206.93 (3,283.57, 5,617.05)			1,266.4 (309.94, 2,285.5)		
Divorced	22	0.46	4,221.69 (3,267.57, 6,377.8)			1,287.2 (286.29, 3,115.59)		
Widowed	11	0.23	3,500.23 (3,072.76, 5,457.83)			545.28 (188.86, 2,044.03)		
Other	197	4.11	3,963.49 (3,011.88, 5,393.92)			1,038.58 (0, 1,848.07)		
Admission route								
Emergency	1,254	26.17	4,221.39 (3,235.89, 5,608.71)	−0.814	0.416	931.93 (0, 1,656.79)	−11.712	< 0.001
Outpatient	3,537	73.83	4,195.16 (3,278.81, 5,586.08)			1,312.24 (410.92, 2,547.94)		
Length of stay (days)								
≤ 3	424	8.85	2,554.84 (1,873.33, 3,477.23)	1,285.907	< 0.001	784.51 (0, 1,233.04)	325.075	< 0.001
4–6	2,237	46.69	3,723.85 (3,006.95, 4,779.72)			1,073.67 (91.2, 1,670.63)		
7–9	1,520	31.73	4,677.37 (3,957.12, 5,786.38)			1,551.7 (685.44, 3,441.05)		
>9	610	12.73	5,989.46 (4,922.52, 7,887.31)			1,791.38 (267.2, 4,377.42)		
Number of other diagnosed diseases								
1–3	122	2.55	3,473.54 (2,518.17, 4,383.86)	207.114	< 0.001	1,982.29 (878.26, 3,741.89)	37.186	< 0.001
4–6	871	18.18	3,841.05 (2,943.42, 5,043.95)			1,274.7 (275.12, 3,071.19)		
7–9	1,504	31.39	4,049.32 (3,196.55, 5,265.61)			1,198.17 (216.35, 2,121.89)		
10–12	1,260	26.30	4,283.42 (3,365.55, 5,775.34)			1,224.33 (141.86, 2,055.67)		
13–15	683	14.26	4,657.36 (3,641.31, 6,094.96)			1,201.16 (185.11, 1,947.08)		
≥16	351	7.33	5,324.28 (4,189.18, 6,801.45)			1,196.74 (181.37, 2,117.6)		
Hypertension								
No	2,244	46.84	4,176.83 (3,225.3, 5,686.08)	−0.88	0.379	1,203.94 (204.33, 2,216.47)	−0.348	0.728
Yes	2,547	53.16	4,221.6 (3,309.94, 5,539.4)			1,235.38 (208.33, 2,243.11)		
Diabetes								
No	3,854	80.44	4,146.81 (3,231.89, 5,522.35)	−3.995	< 0.001	1,248.59 (222.01, 2,319.27)	−2.902	0.004
Yes	937	19.56	4,507.78 (3,457.59, 5,838.97)			1,115.83 (154.33, 1,918.13)		
Surgery level								
No surgery	3,916	81.74	3,926.5 (3,089.99, 4,856.46)	1,344.859	< 0.001	1,150.6 (187.52, 1,867.39)	169.485	< 0.001
Level 1 surgery	131	2.73	5,963.39 (4,557.91, 8,499.92)			1,411.91 (0, 2,236.65)		
Level 2 surgery	117	2.44	5,659 (4,011.61, 6,966.75)			653.38 (0, 2,020.44)		
Level 3 surgery	550	11.48	7,507.38 (6,429.38, 10,569.1)			2,263.83 (674.26, 3,004.68)		
Uncertain level	77	1.61	5,701.85 (4,282.57, 7,789.5)			1,992.1 (1,196.64, 4,541.23)		
Discharge method								
Transfer by doctor's order	105	2.19	4,627.09 (3,590.88, 5,373.28)	42.12	< 0.001	1,145.04 (289.33, 2,163.07)	439.853	< 0.001
Discharge by doctor's order	3,937	82.17	4,188.9 (3,288.89, 5,552.44)			1,167.54 (119.96, 2,012.5)		
Discharge not by doctor's order	503	10.50	4,104.95 (2,919.07, 5,754.31)			1,191.97 (588.03, 1,881.55)		
Death	14	0.29	13,193.04 (5,646.23, 17,650.43)			264.25 (0, 4,917.52)		
Other	232	4.84	4,312.87 (3,379.17, 5,818.08)			4,312.87 (3,379.17, 5,818.08)		

### 4.2 Analysis of influencing factors on hospitalization expenses

#### 4.2.1 Univariate analysis

The univariate analysis results for total hospitalization expenses and out-of-pocket payments of HF patients showed that: total hospitalization expenses were associated with payment method group, type of medical insurance, number of visits, gender, age, length of stay, number of other diagnosed diseases, diabetes, surgery level, and discharge method (*P* < 0.05). Out-of-pocket payments were associated with payment method group, type of medical insurance, number of visits, gender, age, nationality, marital status, admission route, length of stay, number of other diagnosed diseases, diabetes, surgery level, and discharge method (*P* < 0.05; Table 2).

#### 4.2.2 Multiple linear regression

Variables found to be significant (*P* < 0.05) in the univariate analysis were used as independent variables, with log-transformed values of total hospitalization expenses and out-of-pocket payments as dependent variables in multiple linear regression analyses.

The multiple linear regression results showed that: type of medical insurance, number of visits, gender, age (>75 years), length of stay, number of other diagnosed diseases, diabetes, surgery level, and discharge method (discharge by doctor's order, death) were influencing factors for total hospitalization expenses (*P* < 0.05), *F* = 233.95, adjusted *R*^2^ = 0.6114, indicating that all variables in the model explained 61.14% of the variation in total hospitalization expenses. Details are in Table 3.

**Table 3:** Multiple linear regression analysis of factors affecting total hospitalization expenses in HF patients.

Variables	Coef.	Std.Err.	*t*-value	*P*-value	95% Conf.Interval	
Constant	7.633	0.071	107.470	0.000	7.494	7.772
Payment method group (ref = “Non-DRG Period”)						
“Liuzhou DRG Period”	0.038	0.055	0.700	0.487	−0.069	0.146
“Guangxi DRG Period”	−0.031	0.056	−0.550	0.581	−0.140	0.079
Type of medical insurance (ref = Uninsured)						
Urban-Rural Resident Medical Insurance	−0.161	0.018	−8.800	0.000	−0.197	−0.125
Urban Employee Medical Insurance	−0.225	0.022	−10.230	0.000	−0.268	−0.182
Number of visits (ref = 1)						
2	−0.032	0.013	−2.510	0.012	−0.056	−0.007
3	−0.050	0.016	−3.060	0.002	−0.082	−0.018
≥4	−0.060	0.013	−4.580	0.000	−0.085	−0.034
Gender (ref = Male)Female	−0.038	0.010	−4.010	0.000	−0.057	−0.020
Age (ref = 1–60)						
61–65	0.041	0.021	1.960	0.050	0.000	0.081
66–70	0.026	0.018	1.510	0.132	−0.008	0.061
71–75	0.033	0.017	1.930	0.054	−0.001	0.067
76–80	0.039	0.017	2.260	0.024	0.005	0.072
81–85	0.071	0.017	4.150	0.000	0.038	0.105
86–90	0.083	0.021	3.980	0.000	0.042	0.124
>90	0.086	0.031	2.770	0.006	0.025	0.146
Length of stay (days) (ref = ≤ 3)						
4–6	0.385	0.018	22.010	0.000	0.351	0.420
7–9	0.644	0.018	34.850	0.000	0.608	0.680
>9	0.897	0.021	42.490	0.000	0.855	0.938
Number of other diagnosed diseases (ref = 1–3)						
4–6	0.105	0.032	3.310	0.001	0.043	0.167
7–9	0.163	0.031	5.170	0.000	0.101	0.224
10–12	0.221	0.032	6.910	0.000	0.158	0.283
13–15	0.287	0.033	8.640	0.000	0.222	0.352
≥16	0.373	0.035	10.520	0.000	0.304	0.443
Diabetes (ref = yes)	0.067	0.012	5.570	0.000	0.043	0.090
Surgery level (ref = No surgery)						
Level 1 surgery	0.444	0.029	15.470	0.000	0.388	0.500
Level 2 surgery	0.291	0.030	9.580	0.000	0.231	0.350
Level 3 surgery	0.887	0.015	58.740	0.000	0.858	0.917
Uncertain level	0.305	0.038	8.130	0.000	0.231	0.379
Discharge method (ref = Transfer by doctor's order)						
Discharge by doctor's order	−0.069	0.032	−2.180	0.029	−0.132	−0.007
Discharge not by doctor's order	0.028	0.034	0.800	0.423	−0.040	0.095
Death	0.631	0.091	6.910	0.000	0.452	0.810
Other	−0.078	0.059	−1.330	0.183	−0.194	0.037

Out-of-pocket payments were mainly influenced by payment method group (Group 3), type of medical insurance, number of visits (>3), gender, marital status, admission route, length of stay, and surgery level (secondary and tertiary surgeries; *P* < 0.05), *F* = 40.75, adjusted *R*^2^ = 0.2506, indicating that all variables in the model explained 25.06% of the variation in out-of-pocket payments. Details are in Table 4.

**Table 4:** Multiple linear regression analysis of factors affecting out-of-pocket payments in HF patients.

Variables	Coef.	Std.Err.	*t*-value	*P*-value	95% Conf.Interval	
Constant	3.673	0.629	5.840	0.000	2.439	4.907
Payment method group (ref = “Non-DRG Period”)						
“Liuzhou DRG Period”	0.395	0.459	0.860	0.389	−0.504	1.294
“Guangxi DRG Period”	−1.407	0.467	−3.010	0.003	−2.322	−0.491
Type of medical insurance (ref = Uninsured)						
Urban-Rural Resident Medical Insurance	−0.380	0.153	−2.480	0.013	−0.680	−0.080
Urban Employee Medical Insurance	−1.888	0.184	−10.280	0.000	−2.248	−1.528
Number of visits (ref = 1)						
2	−0.165	0.105	−1.570	0.118	−0.371	0.042
3	−0.280	0.137	−2.050	0.040	−0.548	−0.013
≥4	−0.608	0.109	−5.580	0.000	−0.822	−0.395
Gender (ref = Male)Female	0.217	0.080	2.700	0.007	0.059	0.375
Age (ref = 1–60)						
61–65	−0.106	0.174	−0.610	0.542	−0.447	0.235
66–70	−0.201	0.147	−1.370	0.172	−0.489	0.087
71–75	−0.115	0.145	−0.790	0.428	−0.398	0.169
76–80	0.195	0.144	1.360	0.175	−0.087	0.477
81–85	0.334	0.144	2.320	0.020	0.052	0.617
86–90	0.246	0.175	1.410	0.160	−0.097	0.588
>90	0.435	0.259	1.680	0.093	−0.072	0.942
Nationality (ref = Han)						
Zhuang	−0.090	0.097	−0.930	0.355	−0.280	0.101
Other ethnic groups	−2.287	0.635	−3.600	0.000	−3.532	−1.043
Marital status (ref = Single)						
Married	1.945	0.197	9.880	0.000	1.559	2.331
Divorced	2.742	0.599	4.580	0.000	1.568	3.915
Widowed	1.976	0.826	2.390	0.017	0.356	3.596
Other	1.974	0.270	7.300	0.000	1.444	2.504
Admission route (ref = Emergency) Outpatient	1.032	0.088	11.700	0.000	0.859	1.205
Length of stay (days) (ref = ≤ 3)						
4–6	0.593	0.146	4.060	0.000	0.307	0.879
7–9	1.105	0.154	7.160	0.000	0.803	1.408
>9	0.946	0.177	5.340	0.000	0.598	1.294
Number of other diagnosed diseases (ref = 1–3)						
4–6	−0.143	0.265	−0.540	0.588	−0.662	0.375
7–9	−0.099	0.263	−0.380	0.707	−0.615	0.417
10–12	−0.048	0.267	−0.180	0.859	−0.571	0.476
13–15	0.061	0.277	0.220	0.827	−0.483	0.605
≥16	0.210	0.297	0.710	0.479	−0.372	0.793
Diabetes (ref = yes)	−0.053	0.100	−0.540	0.592	−0.249	0.142
Surgery level (ref = no surgery)						
Level 1 surgery	−0.220	0.239	−0.920	0.358	−0.689	0.249
Level 2 surgery	−0.567	0.253	−2.240	0.025	−1.063	−0.071
Level 3 surgery	1.076	0.126	8.530	0.000	0.829	1.324
Uncertain level	0.554	0.313	1.770	0.077	−0.060	1.168
Discharge method (ref = Transfer by doctor's order)						
Discharge by doctor's order	−0.470	0.265	−1.770	0.077	−0.990	0.050
Discharge not by doctor's order	0.257	0.286	0.900	0.369	−0.304	0.819
Death	−0.323	0.763	−0.420	0.672	−1.819	1.173
Other	1.050	0.491	2.140	0.033	0.087	2.013

### 4.3 Analysis of internal structure of hospitalization expenses

#### 4.3.1 Degree of structural change analysis

From 2015 to 2025, the DSV (Calculate using the [Equation 2]) for hospitalization expenses of HF patients in the sample hospital was 59.17%. The DSV was largest from 2022 to 2023 at 68.25%, and smallest from 2016 to 2017 at 4.20%. From 2015 to 2025, the treatment fee had the largest VSV (Calculate using the [Equation 1]) (0.1663), showing a positive change. Diagnosis and material fees generally showed positive but insignificant changes. The drug fee had the smallest VSV (−0.2015), showing a negative change. Comprehensive medical service fees and blood/blood product fees showed negative but insignificant changes. The results are shown in Table 5.

**Table 5:** VSV and DSV of per-admission hospitalization expenses of HF patients in Sample Hospitals from 2015 to 2025.

Time	VSV							DSV/%
	Comprehensive medical service fees	Diagnosis fees	Treatment fees	Drug fees	Blood and blood product fees	Material fees	Other fees	
2015–2016	−0.0051	−0.0298	0.0148	0.0055	0.0082	0.0021	0.0043	6.98
2016–2017	−0.0034	0.0203	−0.0015	−0.0113	0.0006	0.0002	−0.0048	4.20
2017–2018	−0.0443	0.0546	−0.0109	0.0157	−0.0061	0.0019	−0.0109	14.44
2018–2019	−0.0053	0.0230	−0.0165	−0.0114	0.0039	0.0011	0.0052	6.65
2019–2020	0.0136	0.0007	0.0012	−0.0836	0.0050	−0.0029	0.0660	17.31
2020–2021	−0.0258	−0.1226	0.0176	−0.0966	0.0059	0.0288	0.1928	49.01
2021–2022	−0.0061	−0.0296	0.0342	−0.0181	−0.0109	0.0093	0.0212	12.95
2022–2023	0.0739	−0.0543	0.1211	0.0595	−0.0006	0.0868	−0.2863	68.25
2023–2024	0.0145	0.0744	0.0119	−0.0321	−0.0037	−0.0711	0.0060	21.37
2024–2025	−0.1040	0.1181	−0.0056	−0.0291	−0.0045	0.0129	0.0123	28.65
2015–2025	−0.0920	0.0547	0.1663	−0.2015	−0.0023	0.0690	0.0059	59.17

Analysis of the Contribution Rate of Structural Change (CRSV) (Calculate using the [Equation 3]) showed that from 2015 to 2025, comprehensive medical service fees, diagnosis fees, treatment fees, drug fees, and material fees were the main factors affecting structural changes, with a cumulative contribution rate of 98.63%. Among these, the drug fee's CRSV (34.06%) was far greater than other single items, indicating the most significant effect. Looking at consecutive natural years: from 2015 to 2019, diagnosis fees consistently had the largest CRSV; from 2019 to 2020, drug fees had the largest CRSV; from 2021 to 2022, treatment fees had the largest CRSV; from 2023 to 2024 and 2024 to 2025, diagnosis fees had the largest CRSV. The results are shown in Table 6.

**Table 6:** CRSV of per-admission hospitalization expenses of HF patients in Sample Hospitals from 2015 to 2025 (%).

Time	Comprehensive medical service fees	Diagnosis fees	Treatment fees	Drug fees	Blood and blood product fees	Material fees	Other fees
2015–2016	7.29	42.71	21.23	7.91	11.70	3.06	6.10
2016–2017	8.09	48.25	3.66	26.76	1.36	0.39	11.48
2017–2018	30.68	37.80	7.56	10.89	4.21	1.32	7.55
2018–2019	8.04	34.59	24.76	17.20	5.93	1.63	7.85
2019–2020	7.84	0.42	0.70	48.30	2.88	1.70	38.15
2020–2021	5.25	25.03	3.58	19.72	1.20	5.87	39.35
2021–2022	4.74	22.88	26.44	13.95	8.43	7.17	16.39
2022–2023	10.83	7.95	17.74	8.72	0.10	12.71	41.95
2023–2024	6.78	34.81	5.59	15.01	1.73	33.25	2.82
2024–2025	36.30	41.21	1.95	10.17	1.58	4.49	4.30
2015–2025	15.55	9.25	28.11	34.06	0.39	11.66	0.99

#### 4.3.2 Novel gray relational analysis

The correlation coefficients, relational degrees, and relational sequences (Calculate using the ([Equations 4–6]) for various expense items in the average per-admission hospitalization expenses of HF patients from 2015 to 2025 are shown in Table 7. Among all hospitalization expense items across the years, diagnosis fees had the largest correlation coefficient. Over the 11-year period, the top two items most closely associated with the average per-admission hospitalization expenses were diagnosis fees (0.8797) and drug fees (0.7647), followed by comprehensive medical service fees, treatment fees, other fees, and material fees. Blood and blood product fees had the smallest relational degree (0.6559).

**Table 7:** Correlation coefficients, relational degrees, and relational sequences of per-admission hospitalization expenses of HF patients in sample hospitals from 2015 to 2025.

Fees	Correlation coefficients											Relational degrees	Correlation sequences
	2015	2016	2017	2018	2019	2020	2021	2022	2023	2024	2025		
Comprehensive medical service fees	0.7164	0.7245	0.7890	0.7605	0.7789	0.7609	0.6902	0.6270	0.7020	0.7802	0.7178	0.7316	3
Diagnosis fees	0.8526	0.8418	0.9239	0.9603	1.0000	0.9760	0.8236	0.7406	0.7471	0.8659	0.9444	0.8797	1
Treatment fees	0.6294	0.6459	0.7096	0.6995	0.7131	0.6893	0.6403	0.5958	0.6894	0.7657	0.7535	0.6847	4
Drug fees	0.7794	0.7938	0.8540	0.8584	0.8724	0.7976	0.6892	0.6206	0.6873	0.7411	0.7177	0.7647	2
Blood and blood product fees	0.6211	0.6347	0.6988	0.6910	0.7132	0.6910	0.6371	0.5746	0.6111	0.6768	0.6660	0.6559	7
Material fees	0.6231	0.6343	0.6982	0.6937	0.7147	0.6892	0.6448	0.5900	0.6658	0.7021	0.6987	0.6686	6
Other fees	0.6255	0.6375	0.6994	0.6896	0.7123	0.7167	0.7568	0.7067	0.6081	0.6777	0.6738	0.6822	5

## 5 Discussion

### 5.1 DRG payment can effectively reduce hospitalization expenses for HF patients

Based on the quartiles of hospitalization expenses by “Payment Method Group,” the total hospitalization expenses for HF patients showed an overall declining trend after DRG implementation. This indicates that the DRG policy helped reduce hospitalization expenses. The reason is that China's medical insurance bureau pays hospitals a “standard” fee for each DRG group. Under the settlement principle of “keeping the surplus, not compensating for overruns,” any excess cost beyond the standard is borne by the hospital, incentivizing hospitals to actively reduce costs and control medical expenses (22). This finding is consistent with Chen Fenglei's research (23). The payment method group (Group 3) was a main influencing factor for out-of-pocket payments. The quartiles for out-of-pocket payments significantly decreased sequentially under the Liuzhou (municipal) and Guangxi (provincial) DRG policies. Under the DRG policy, the quartiles for out-of-pocket payments were significantly lower overall than those for total hospitalization expenses. Out-of-pocket payments for non-DRG patients were also significantly higher than for other insured patients, indicating that the DRG policy effectively reduced patients' out-of-pocket payments, alleviating their economic disease burden. This suggests a positive policy orientation for DRG, aligning with research by Wu Ni, it also reminds medical institutions to seek a balance between cost control and medical quality, avoiding situations where medical quality is compromised in order to control medical expenses, and to effectively reduce the burden on patients while providing high-quality, safe, and effective medical services (17).

### 5.2 Medical insurance type, number of visits, hospital length of stay, surgery level, and gender affect both total hospitalization expenses and out-of-pocket payments

The results of this study show statistically significant differences in both total hospitalization expenses and out-of-pocket payments based on medical insurance type, number of visits, length of stay, surgery level, and gender. The quartiles for hospitalization expenses of HF patients enrolled in Urban-Rural Resident Basic Medical Insurance and Urban Employee Basic Medical Insurance were also significantly lower than those for uninsured patients, indicating that having medical insurance significantly reduces the medical burden for HF patients. Generally, a higher number of visits, longer length of stay, and higher surgery level were associated with lower hospitalization expense quartiles. More visits suggest disease recurrence, worsening condition, and increased treatment difficulty, leading to higher costs for examinations and treatments. A longer length of stay positively contributes to increased hospitalization expenses, directly adding to medical costs (24). Surgical treatment for HF increases patients' direct medical costs, such as one-time surgical medical material fees. Higher surgery levels may be associated with higher-risk disease types, more expensive surgical equipment and materials, more complex treatment measures, and relatively higher medical expenses (12). This study found a statistically significant difference in hospitalization expenses between males and females. Comparison of median total hospitalization expenses showed that males had higher costs than females. Differences in physiological structure and metabolic characteristics between men and women lead to variations in disease types and treatment responses, affecting patients' healthcare-seeking behavior and medical needs (25). Chinese males are generally more exposed and for longer durations to risk factors such as smoking and alcohol consumption compared to females (17), and the severity of illness in males is generally higher than in females, leading to differentiated hospitalization expenses.

### 5.3 The impact of other factors on total hospitalization Expenses and out-of-pocket Payments for heart failure differs

Statistically significant differences were found in total hospitalization expenses based on age, number of other diagnosed diseases, diabetes, and discharge method, but not in out-of-pocket payments. Compared to the group under 60 years old, patients over 76 had higher hospitalization expenses. Degeneration of bodily functions in the older adults leads to more complex treatment processes, slower recovery, and poorer prognosis, requiring more inpatient treatment (12). However, medical insurance effectively reduces the burden of out-of-pocket hospitalization expenses for patients across different age groups. A higher number of other diagnosed diseases and having diabetes were associated with higher median total hospitalization expenses for HF patients. Greater disease severity and complexity consume more medical resources during treatment (26), correspondingly increasing total hospitalization expenses. The lack of impact on out-of-pocket payments suggests that medical insurance policy support reduces the disease burden from other conditions for HF patients. Regarding discharge methods, patients discharged by doctor's order or who died had higher hospitalization expenses, possibly due to disease complexity, additional treatments or resuscitations increasing medical expenditures (11). While discharge for recovery or death settlement affects total hospitalization expenses, most costs are reimbursed according to medical insurance, so out-of-pocket payments are not affected.

### 5.4 Treatment, diagnosis, and drug fees are the main factors in the internal changes of hospitalization expenses

The structural change analysis results show that treatment fees exhibited a positive change, with their proportion significantly increasing. Drug fees showed a negative change, with their proportion significantly decreasing. Drug fees had the largest Contribution Rate of Structural Variation (CRSV), indicating the greatest magnitude of change and being the primary factor driving fluctuations in the hospitalization expense structure. The novel gray relational analysis results indicate that diagnosis and drug fees had the greatest impact on the average per-admission hospitalization expenses, being most closely associated with them. This suggests that treatment, diagnosis and drug fees remain key factors influencing hospitalization expenses, a result similar to existing research conclusions (27). The reasons are speculated as follows: the impact of diagnosis fees on hospitalization expenses results from multiple factors. Technologically, accurate diagnosis of cardiac diseases often relies on high-cost precision tests like echocardiography and coronary angiography, whose equipment and material costs directly drive up diagnosis fees. Within the medical system, there might be a degree of “compensating for medical services with technology” phenomenon in healthcare institutions (18), adding to diagnosis fee costs. Treatment fees have shown a reasonable upward trend in recent years, reflecting the technical value of medical personnel. This helps realign medical service prices to reflect healthcare workers' labor value, fostering a benign incentive mechanism that prioritizes medical technology and promotes sustainable, healthy development of the medical system. Furthermore, with the gradual implementation of the DRG payment system, the hospital has achieved certain results in controlling drug and material costs, primarily due to a series of national healthcare reform policies such as abolishing drug and material markup and centralized volume-based procurement. Simultaneously, the hospital strengthened supervision over rational drug use and high-value medical consumables (28), effectively reducing drug expenses and their proportion in hospitalization expenses. This leads to reasonable control of medical costs and alleviates patients' economic burden. Additionally, the implementation of DRG payment has encouraged further standardization of clinical diagnosis and treatment practices, reducing irrational drug use and over-treatment during therapy. Related research also points out that promoting DRG reform is an inevitable path to control unreasonable growth in medical expenses and improve the efficiency of medical insurance fund use (29).

## 6 Conclusions

This study analyzed the influencing factors and structural changes in hospitalization expenses for 4,791 HF inpatients at a secondary Grade-A hospital in Liuzhou City under the DRG payment system reform. The results indicate that the DRG payment system can effectively reduce hospitalization expenses for HF patients. Medical insurance type, number of visits, length of stay, surgery level, and gender affect both total hospitalization expenses and out-of-pocket payments. Age, number of other diagnosed diseases, diabetes, and discharge method influence total hospitalization expenses but not out-of-pocket payments. Treatment, diagnosis, and drug fees are the main factors in the internal structural changes of hospitalization expenses. Under the DRG payment system reform, hospitals should establish performance evaluation mechanisms compatible with the DRG model, standardize treatment protocols, monitor non-compliant diagnosis and treatment behaviors, improve medical insurance reimbursement policies, and control medical service costs. Under DRG, hospitals should fully utilize the cost advantages brought by centralized procurement to reduce hospitalization expenses like drug fees, providing high-quality and efficient medical services to patients under refined management (30).

This study has three limitations. First, existing research has not fully considered the interactions among various influencing factors, making it difficult to clarify the complex relationships between multiple factors and hospitalization expenses. Furthermore, the data source for influencing factors is limited to the front page of inpatient medical records, making the analysis of influencing factors incomplete. Second, the study data only included HF patient samples from one hospital in Liuzhou City, and the sample size and case sources are insufficient to demonstrate generalizability. The impact of economic level differences across regions on the results was not considered, which may limit applicability to areas with different economic conditions. Third, the sample hospital is a secondary Grade-A hospital, whereas higher-level hospitals tend to admit more severe cases, and the burden of medical expenses is more significantly affected by medical insurance payment methods. Future research should continue to track the impact of DRG payments on hospitalization expenses for HF across different hospital levels, conducting studies with longer time spans and larger sample sizes.

## Data Availability

The original contributions presented in the study are included in the article/supplementary material, further inquiries can be directed to the corresponding author.

## References

[1] Chinese Chinese Society of Cardiology Chinese Medical Doctor Association Cardiovascular Physicians Branch Chinese Medical Doctor Association Heart Failure Committee Editorial Editorial Board of Chinese Journal of Cardiology. Chinese guidelines for the diagnosis and treatment of heart failure 2024. Chin J Cardiol. (2024) 52:235–75. doi: 10.3760/cma.j.cn112148-20231101-00405

[2] Chen QF Chen L Katsouras CS Liu C Shi J Liang D . Global burden of heart failure and its underlying causes in 204 countries and territories, 1990-2021. Eur Heart J Qual Care Clin Outcomes. (2025) 11:493–509. doi: 10.1093/ehjqcco/qcae110 39774847

[3] Chen J Aronowitz P. Congestive heart failure. Med Clin North Am. (2022) 106:447–58. doi: 10.1016/j.mcna.2021.12.002 35491065

[4] Kansal AR Cowie MR Kielhorn A Krotneva S Tafazzoli A Zheng Y . Cost-effectiveness of ivabradine for heart failure in the United States. J Am Heart Assoc. (2016) 5:e003221. doi: 10.1161/JAHA.116.003221 27153871 PMC4889192

[5] Chan YK Tuttle C Ball J Teng TK Ahamed Y Carrington MJ . Current and projected burden of heart failure in the Australian adult population: a substantive but still ill-defined major health issue. BMC Health Serv Res. (2016) 16:501. doi: 10.1186/s12913-016-1748-0 27654659 PMC5031369

[6] National Center for Cardiovascular Diseases Shengshou H. Report on cardiovascular health and diseases in China 2024: an updated summary. Chin Circ J. (2025) 40:521–59. doi: 10.3969/j.issn.1000-3614.2025.06.001

[7] Heidenreich P. Heart failure management guidelines: new recommendations and implementation. J Cardiol. (2024) 83:67–73. doi: 10.1016/j.jjcc.2023.10.009 37949313

[8] Darbà J Ascanio M Rodríguez A Charman SJ Okwose NC Stefanetti RJ . Economic burden of heart failure in Europe: a systematic review of costs and cost-effectiveness. ESC Heart Fail. (2025) 12:4055–68. doi: 10.1002/ehf2.70017 41296502 PMC12719840

[9] Yuanyuan L Changping L Zhuang C Hui K Chunhua S Bao Z . Hospitalization expense and its influencing factors in heart failure patients with medical insurance. Chin J Public Health. (2013) 29:313–5.

[10] Yanan P Qian Z Xintao S Jing Y. Analysis of hospitalization costs for heart failure patients based on a decision tree model. Chin J Health Inform Manag. (2024) 21:920–9. doi: 10.3969/j.issn. 1672-5166.2024.06.021

[11] Panpan B Tiane L Huanzhen C. Analysis of factors affecting hospitalization costs in heart failure patients based on structural equation modeling. Chin J Integr Med Cardio-Cerebrovasc Dis. (2023) 21:1505–9. doi: 10.12102/j.issn.1672-1349.2023.08.029

[12] Wei T Meiqi Z Shuaifei W Xin W Xiudan P. Analysis of hospitalization costs and influencing factors in 233 patients with heart failure. Chin Med Rec. (2021) 22:64–7.

[13] Ye L Bowen L. Study on DRG cost of 564 heart failure cases in Tertiary Grade A hospitals in 2020. Jiangsu Health Syst Manag. (2022) 33:1514–7.

[14] García-Quintana A Alonso Ramos H Parrondo J. [Translated article] Cost-utility of sacubitril/valsartan in heart failure with reduced ejection fraction in Spain. Farm Hosp. (2025) 49:T359–66. doi: 10.1016/j.farma.2025.08.004 40849246

[15] Psotka MA Fonarow GC Allen LA Joynt Maddox KE Fiuzat M Heidenreich P . The hospital readmissions reduction program: nationwide perspectives and recommendations: a JACC: Heart Failure Position Paper. JACC Heart Fail. (2020) 8:1–11. doi: 10.1016/j.jchf.2019.07.012 31606360

[16] Xiaoping S. Study on impact of DRG payment mode reform on the medical expense burden of hospitalized patients. Health Econ Res. (2025) 42:61–4. doi: 10.14055/j.cnki.33-1056/f.2025.01.021

[17] Ni W Xiaoyuan Z Yufan W Mingzheng T Luo W. Study on influencing factors of hospitalization costs and days for patients with cardiovascular and cerebrovascular diseases in Sichuan Province Under DRG Payment. Med Soc. (2024) 37:77–84. doi: 10.13723/j.yxysh.2024.10.012

[18] Yunsha W Jinfeng T Yufan W Zhao Z Xiaoyuan Z. Comparative study on the control of hospitalization costs of cardiovascular disease patients under DRG and DIP payment. Health Econ Res. (2025) 42:67–70. doi: 10.14055/j.cnki.33-1056/f.2025.03.022

[19] Liwen L Lifeng H Wenjie Z Yuyu W Lu Z Ying X . Influencing factors of hospitalization costs for colon cancer patients based on gradient boosting machine in Zhuhai. Med Soc. (2025) 38:25–31. doi: 10.13723/j.yxysh.2025.03.004

[20] Fengqin L Liqi T Jinfeng J. Analysis on the hospitalization expenses of cervical cancer patients in a Grade A tertiary hospital of based on structural change degree and grey correlation. Chin Hosp Manag. (2022) 42:74–7.

[21] Wei Z Shuangling Q. A new grey correlation analysis on inpatient costs for malignant tumor patients based on DRG. Health Econ Res. (2022) 39:50–2+57. doi: 10.14055/j.cnki.33-1056/f.2022.04.012

[22] Weidong D Xuange Z Xin Y. Effectiveness and governance experience of DRG payment reform in Quzhou City. Health Econ Res. (2023) 40:8–11. doi: 10.14055/j.cnki.33-1056/f.2023.11.003

[23] Fenglei C Bing L Jing Q Nenghai B Ying Z Dunke J . Research on the changes in patients' hospitalization expenses after the DRG payment reform in a Grade III Level A hospital in Guangxi. Chin Hosp. (2023) 27:53–5. doi: 10.19660/j.issn.1671-0592.2023.01.14

[24] Lang T Shulei L Ruoxi F Jingxin Y Xiaoyuan Z. Prediction of hospitalization expenses for ischemic heart disease: machine learning based on SHAP theory. Chin Jo Evid Based Med. (2025) 25:881–7. doi: 10.7507/1672-2531.202503190

[25] Rong T Hong M Jingyi X Yuanyuan D Yue L. Research on influencing factors of hospitalization expenses of type 2 diabetes based on BP neural network. Chin Med Rec. (2022) 23:63–7.

[26] Duman H Çetin M Durakoglugil ME Degirmenci H Hamur H Bostan M . Relation of angiographic thrombus burden with severity of coronary artery disease in patients with ST segment elevation myocardial infarction. Med Sci Monit. (2015) 21:3540–6. doi: 10.12659/MSM.895157 26573108 PMC4655613

[27] Zhen H Yuting Z Ping L Juan X. Prediction and analysis of changes in hospitalization medical cost structure for chronic heart failure based on a combined Markov Chain and GM(1,1) Model. Chongqing Med J. (2025) 54:243–6.

[28] Yongyuan Z Qingliang W Xuehong L. Structural changes and influencing factors of hospitalization expenses for rare diseases patients in a tertiary general hospital in Shandong Province. J Shandong Univ. (2025) 63:111–9. doi: 10.6040/j.issn.1671-7554.0.2024.0793

[29] Qiongqiong Z Yulei S Ye W Pei C Yilin C Bei W . Analysis of inpatient cost structure and influencing factors for ICU patients with respiratory diseases under DRG payment. Chin Hosp. (2025) 29:73–6. doi: 10.19660/j.issn.1671-0592.2025.5.16

[30] Qiong D Yong G Jiahui H. Study on the impact of DRG payment on hospitalization costs and services for patients with osteoporotic vertebral fractures in a hospital. Chin Hosp. (2025) 29:80–3. doi: 10.19660/j.issn.1671-0592.2025.11.19

